# Supplementary material for: The well-being and work-related stress of senior school leaders in Wales and Northern Ireland during COVID-19 “educational leadership crisis”: A cross-sectional descriptive study
Source: PLoS One. 2024 Apr 10;19(4):e0291278. doi: 10.1371/journal.pone.0291278 (PMC11006137; doi:10.1371/journal.pone.0291278)
Supplement: S1 Fig — (DOCX) [file pone.0291278.s002.docx]

| **Category** | **Sub-category** |
| --- | --- |
| **Socio-demographic information** | Gender* |
|  | Age |
|  | Type of school* |
|  | Position* |
|  | Weekly teaching load* |
|  | Weekly working hours* |
|  | Assessment of working time |
|  | Number of students* |
|  | Socioeconomic status* |
|  | Participation in the state programme on health and education |
|  | Media concept at school |
|  | Media equipment at school |
|  | Quality of and capacity regarding the school’s digital media infrastructure |
| **Current work situation** | Sense of Coherence* |
|  | Perceived stress* |
|  | Self-endangering behaviour* |
|  | Work satisfaction |
| **Health information in the context of the COVID-19 pandemic** | Information satisfaction on coronavirus |
|  | Confusion due to information on coronavirus |
|  | Vaccination readiness coronavirus |
|  | Attitudes about vaccination |
|  | Corona-specific health literacy |
| **Health promotion and prevention in school** | Health promoting school factors |
| **Health situation** | Perceived health needs of pupils |
|  | Perceived health needs of teachers |
|  | General health* |
|  | Chronic disease |
|  | Impairment by health problems |
|  | Well-being* |
|  | Exhaustion* |
|  | Psychosomatic complaints * |

* denounces sub-categories used in analyses
