# Supplementary material for: The well-being and work-related stress of senior school leaders in Wales and Northern Ireland during COVID-19 “educational leadership crisis”: A cross-sectional descriptive study
Source: PLoS One. 2024 Apr 10;19(4):e0291278. doi: 10.1371/journal.pone.0291278 (PMC11006137; doi:10.1371/journal.pone.0291278)
Supplement: S3 Fig — (PDF) [file pone.0291278.s004.pdf]

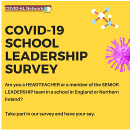

COVID-19 School Leadership Survey - Northern Ireland

This survey is part of an international project focussing on school headteachers and senior leadership staff views on work-related stress, the use of health-related information, and health and wellbeing in the context of the COVID-19 pandemic.

* Required

1

I have read the information sheet (version 1, dated 5/5/21) about taking part in the study. I have had the opportunity to consider the information, ask questions and have had these answered satisfactorily. I understand that my participation in this research is voluntary and that I am free to withdraw at any time without giving any reason. I am happy to take part in this online survey. *

You can read the information sheet here: https://ncphwr.org.uk/wp-content/uploads/2021/12/COVID-19-HL-Information-sheet-lb50.pdf

◯ Yes

◯ No

2

I would be happy for my anonymised responses to be shared with findings from the international study (COVID-HL network) *

- ◯ Yes
- ◯ No

About You

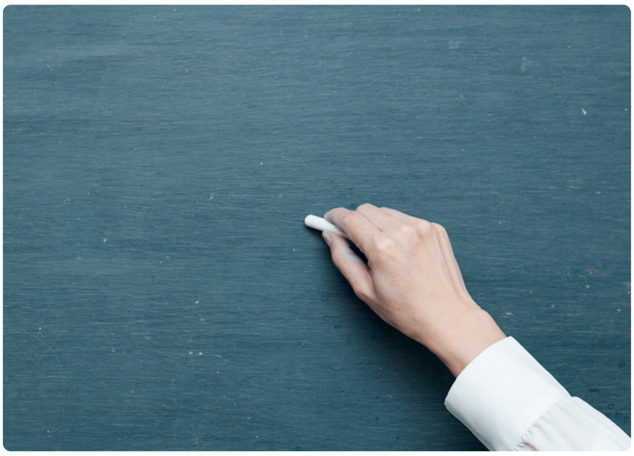

Let's get started by asking you some information about yourself.

3

What country do you work in?

- ○ England
- ○ Northern Ireland

4

What is your ethnic group?

- White
- Mixed / multiple ethnic groups
- Asian / Asian British
- Black / African / Caribbean / Black British
- Irish Traveller
- Prefer not to say

5

If other, please state

6

Please indicate your sex

- ◯ Male
- ◯ Female
- ◯ Prefer not to say
- ◯ Other

7

If other, how do you identify

8

How old are you

- ◯ 39 and below
- ◯ 40-49
- ◯ 50-59
- ◯ 60+

Your Health

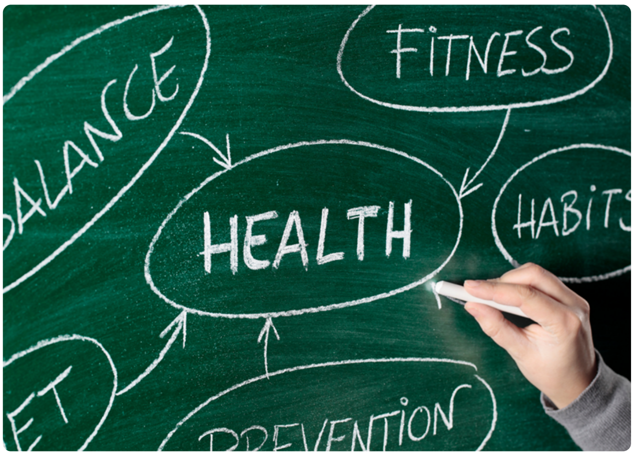

Now we'd like to ask some questions about your general health.

9

Please indicate for each of the five statements which is closest to how you have been feeling over the last two weeks.

	All of the time	Most of the time	More than half of the time	Less than half of the time	Some of the time	None of the time
I have felt cheerful and in good spirits	○	○	○	○	○	○
I have felt calm and relaxed	○	○	○	○	○	○
I have felt active and vigorous	○	○	○	○	○	○
I woke up feeling fresh and rested	○	○	○	○	○	○
My daily life has been filled with things that interest me	○	○	○	○	○	○

10

How is your health in general

- ◯ Very good
- ◯ Good
- ◯ Moderate
- ◯ Bad
- ◯ Very bad

11

Do you suffer from a chronic disease or a long-lasting health problem (this refers to diseases or health problems that last or are expected to last at least 6 months)

(this refers to diseases or health problems that last or are expected to last at least 6 months)

- ◯ Yes
- ◯ No

12

To what extent are you impaired by your chronic illness in activities of normal everyday life?

- ◯ Not at all impaired
- ◯ Moderately impaired
- ◯ Strongly impaired

13

How often do you suffer from the following health symptoms?

	Never	Rarely	Sometimes	Often	Always
Palpitations and/or chest pain	◯	◯	◯	◯	◯
Stomach and/or intestinal complaints	◯	◯	◯	◯	◯
Headaches	◯	◯	◯	◯	◯
Muscle pain (e.g. neck, shoulder, back)	◯	◯	◯	◯	◯

Your School

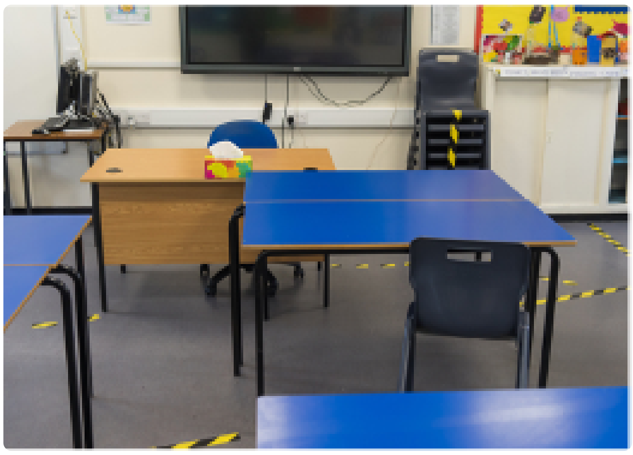

Please provide details here about the school you work in.

14

What region is your school in?

- ◯ South East England
- ◯ South West England
- ◯ North West England
- ◯ North East England
- ◯ East of England
- ◯ East Midlands
- ◯ West Midlands
- ◯ Yorkshire and Humberside
- ◯ Greater London
- ◯ Antrim
- ◯ Armagh
- ◯ Down
- ◯ Fermanagh
- ◯ Londonderry
- ◯ Tyrone

15

Is your school primary or secondary?

- ◯ Primary school (4-11 year olds)
- ◯ Secondary/Post primary (11-16+)

16

What type of school do you work in?

Select all that apply

- [ ] Special school
- [ ] Private or independent school
- [ ] Pupil Referral Unit
- [ ] Controlled
- [ ] Voluntary
- [ ] Controlled integrated
- [ ] Specialist teaching facility within primary school
- [ ] Academy
- [ ] Federation
- [ ] Grant Maintained Integrated
- [ ] Independent
- [ ] Catholic Maintained

17

How many pupils are in your school?

The value must be a number

18

What percentage of pupils in your school are eligible for free school meals?

- ◯ 0% - 20%
- ◯ 21% - 40%
- ◯ 41% - 60%
- ◯ 61% - 80%
- ◯ 81% - 100%
- ◯ Do not know

19

What percentage of pupils in your school come from families from lower social classes

This is about the socioeconomic status of your pupils

The value must be a number

20

What percentage of pupils in your school come from families from middle social classes

This is about the socioeconomic status of your pupils

The value must be a number

21

What percentage of pupils in your school come from families from higher social classes

This is about the socioeconomic status of your pupils

The value must be a number

22

Does your school participate in the Network of Healthy Schools scheme?

- ◯ Not part of the Healthy Schools scheme
- ◯ Yes, for less than 1 year
- ◯ Yes, for 1-2 years
- ◯ Yes, for 2-3 years
- ◯ Yes, for 3 years or more
- ◯ Not sure

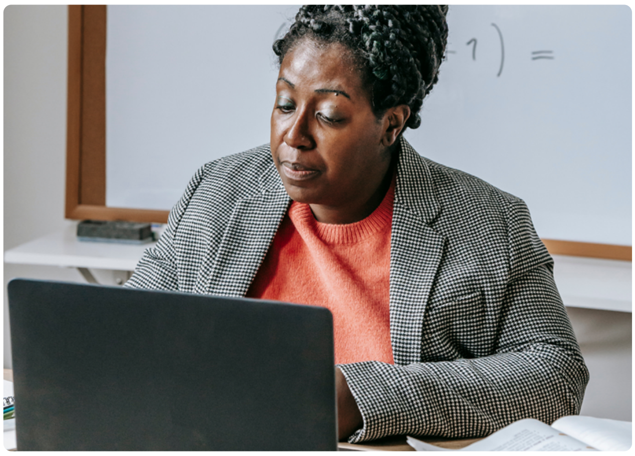

Your Role

This section is about your role in your school.

23

What is your role?

- ◯ Headteacher
- ◯ Deputy Headteacher
- ◯ Senior leadership

24

If senior leadership, please describe your role

25

How many years have you been working in a senior leadership position?

The value must be a number

26

What is your weekly teaching load?

Please indicate the number of school hours per week.

The value must be a number

27

How many total hours do you currently work on average per week?

This includes management and leadership tasks, preparation and follow-up, class time, correction, extracurricular activities, etc.

The value must be a number

28

What are your current weekly working hours like compared to pre-COVID in March 2020?

- ◯ Lower than before the COVID-19 pandemic
- ◯ About the same
- ◯ Higher than before the COVID-19 pandemic

Your current working situation and demands

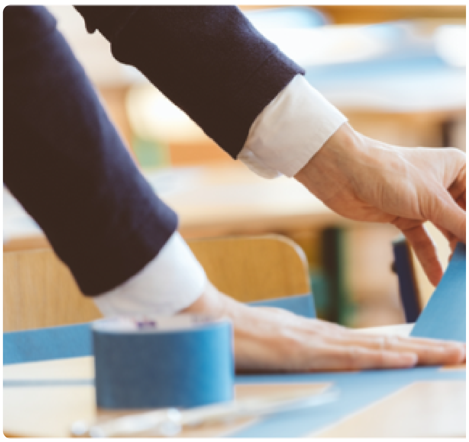

How do you find your current work situation in general?

29

For these questions, please indicate your current feeling about your work using the scale below.

Unmanageable ☆ ☆ ☆ ☆ ☆ ☆ ☆ Manageable

30

Meaningless ☆ ☆ ☆ ☆ ☆ ☆ ☆ Meaningful

31

Unstructured ☆ ☆ ☆ ☆ ☆ ☆ ☆ Structured

32

Impossible to influence ☆ ☆ ☆ ☆ ☆ ☆ ☆ Easy to influence

33

Insignificant ☆ ☆ ☆ ☆ ☆ ☆ ☆ Significant

34

Unclear ☆ ☆ ☆ ☆ ☆ ☆ ☆ Clear

35

Uncontrollable ☆ ☆ ☆ ☆ ☆ ☆ ☆ Controllable

36

Unrewarding ☆ ☆ ☆ ☆ ☆ ☆ ☆ Rewarding

37

Unpredictable ☆ ☆ ☆ ☆ ☆ ☆ ☆ Predictable

38

The following statements are related to your work situation and how you experience this situation

	Never	Rarely	Sometimes	Often	Always
At work I feel mentally exhausted	○	○	○	○	○
After a day at work, I find it hard to recover my energy	○	○	○	○	○
At work, I feel physically exhausted	○	○	○	○	○

The questions in this scale are about how you have found your work in the last month.

In the last month how often have you...

	Never	Almost never	Sometimes	Fairly often	Very often
Been upset because of something that happened unexpectedly?	◯	◯	◯	◯	◯
Felt that your were unable to control the important things?	◯	◯	◯	◯	◯
Felt nervous and "stressed" at school?	◯	◯	◯	◯	◯
Felt confident about your ability to handle your professional work-related problems caused by the COVID-19 pandemic?	◯	◯	◯	◯	◯
Found that you could not cope with all your work tasks?	◯	◯	◯	◯	◯
Been able to control irritations at your work?	◯	◯	◯	◯	◯
Felt that you were on top of things at work?	◯	◯	◯	◯	◯

Been angered because of things that were outside of your control at work?	○	○	○	○	○
Felt difficulties at work were piling up so high that you could not deal with them?	○	○	○	○	○
Felt that things at work were going your way?	○	○	○	○	○

The questions in this scale are about how you have found your work in the last three months.

In the last three months, how often have you...

	Never	Almost never	Sometimes	Fairly often	Very often
Been available for your colleagues, pupils and parents in your free time?	○	○	○	○	○
Given up leisure activities in favour of work?	○	○	○	○	○
Forgone getting sufficient sleep in favour of work?	○	○	○	○	○
Worked extra hours in your free time (after work, during holidays, at weekends, on public holidays)?	○	○	○	○	○
Waived breaks (short or lunch break) during your working hours?	○	○	○	○	○
Worked longer than contractually agreed?	○	○	○	○	○

In the past three months, how often have you worked at a pace that...

	Never	Almost never	Sometimes	Fairly often	Very often
You find burdensome	◯	◯	◯	◯	◯
You cannot sustain in the long term	◯	◯	◯	◯	◯
You know is not good for you	◯	◯	◯	◯	◯

In the past three months, how often have you been required to...

	Never	Almost never	Sometimes	Fairly often	Very often
Be satisfied with a lower quality of work then your normally would be?	○	○	○	○	○
Lower your own work output standard?	○	○	○	○	○
Carry out more superficial work (e.g. clerical tasks, admin)?	○	○	○	○	○

Your school's digital provision

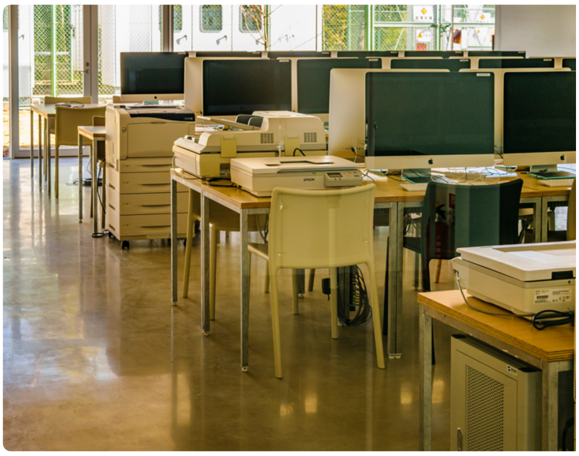

Here, we'd like to know about the digital provision of equipment and resources in your school.

43

Does your school use a specific media literacy curriculum and action plan that regulates the use of digital media (e.g. equipment, software) as well as the teaching of digital education and literacy?

- [ ] Yes
- [ ] No
- [ ] Not sure

Does your school have:

	Yes, a sufficient number	Yes, but not enough	No
Laptops as a permanent media pool in classrooms	○	○	○
Laptops as a mobile media pool (e.g. on laptop trolleys)	○	○	○
Tablets as a permanent media pool in classrooms	○	○	○
Tablets as a mobile media pool (e.g. on tablet trolleys)	○	○	○
Smartboards in classrooms	○	○	○
Projectors as a permanent feature of classrooms (i.e. ceiling projectors)	○	○	○
Mobile projectors	○	○	○
Pupil own devices, including laptops and mobiles in Learning and Teaching	○	○	○

How would you rate the (digital) media equipment and infrastructure at your school overall regarding the following aspects?

	1 (very good)	2	3	4	5	6 (very bad)
The adequacy of the IT and media infrastructure to meet the requirements of COVID-19 related home schooling	○	○	○	○	○	○
The competencies and skills of the school staff to handle the IT and media infrastructure	○	○	○	○	○	○
The quality and stability of the internet connection at your school	○	○	○	○	○	○

Pupil and school staff health

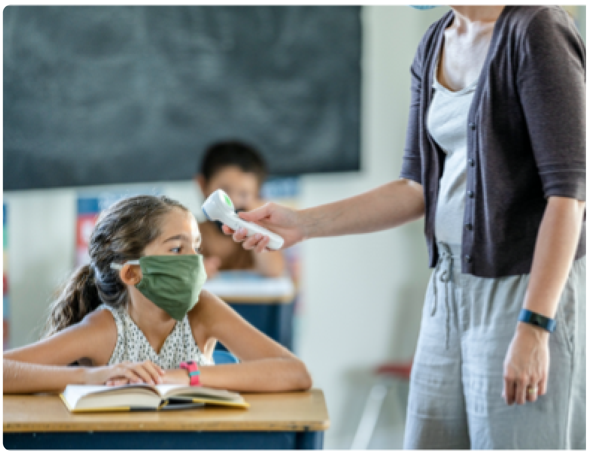

These questions are about the health and wellbeing of pupils and staff in your school.

Please rate how important you think the following health issues are for your PUPILS since the outbreak of the COVID-19 pandemic in March 2020.

Importance is rated higher the more frequently these problems occur in everyday school life

	Very low	Low	High	Very high
Stress and coping	○	○	○	○
Internalised problems (e.g. anxiety, depression)	○	○	○	○
Behavioural problems (e.g. bullying)	○	○	○	○
Substance use (e.g. alcohol, tobacco)	○	○	○	○
Media use	○	○	○	○
Overweight	○	○	○	○
Healthy eating	○	○	○	○
Physical activity/sport	○	○	○	○

Please rate how important you think the following health issues are for your STAFF since the outbreak of the COVID-19 pandemic in March 2020.

Importance is rated higher the more frequently these problems occur in everyday school life

	Very low	Low	High	Very high
Stress and coping	○	○	○	○
Internalised problems (e.g. anxiety, depression)	○	○	○	○
Behavioural problems (e.g. bullying)	○	○	○	○
Substance use (e.g. alcohol, tobacco)	○	○	○	○
Media use	○	○	○	○
Overweight	○	○	○	○
Healthy eating	○	○	○	○
Physical activity/sport	○	○	○	○

School health promotion and prevention activities in the context of COVID-19

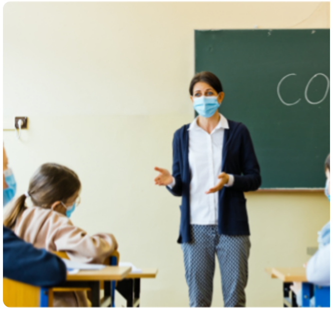

We'd like to find out about school health promotion and prevention activities that are implemented in your school.

How true are the following statements for your school?

At our school...

	Not true at all	Mostly not true	Somewhat true	Totally true
Pupils are taught basic information about COVID-19 (e.g. causes of its development, spread)	○	○	○	○
Pupils learn ways to protect themselves from infection.	○	○	○	○
Pupils learn how to get enough exercise despite the restrictions due to COVID-19.	○	○	○	○
Pupils learn how to eat healthily despite restrictions due to COVID-19.	○	○	○	○
Pupils are supported in dealing with worries and fears caused by COVID-19.	○	○	○	○
Spaces (including digital) of social interaction and exchange [illegible]	○	○	○	○

are created despite the COVID-19 related restrictions.

There is a consensus that health and school performance of pupils are interrelated.	◯	◯	◯	◯

How true are the following statements for your school?

At our school...

	Not true at all	Mostly not true	Somewhat true	Totally true
School staff are supported in dealing with stressful situations caused by COVID-19 (e.g. stress).	○	○	○	○
Health-promoting aspects play an important role in the design of teaching and learning conditions (including home schooling).	○	○	○	○
Health promoting aspects play an important role in the design of working conditions (including home office)	○	○	○	○
There are regular further training courses on health-related topics (protection against infection with COVID-19, dealing with stressed pupils).	○	○	○	○

How true are the following statements for your school?

At our school...

	Not true at all	Mostly not true	Somewhat true	Totally true
Pupils are involved in the planning of prevention and health promotion activities.	○	○	○	○
We work closely with parents when it comes to promoting and protecting children's health.	○	○	○	○
We work closely with community stakeholders from the health and social sectors when it comes to promoting the health of our pupils.	○	○	○	○

Information about COVID-19

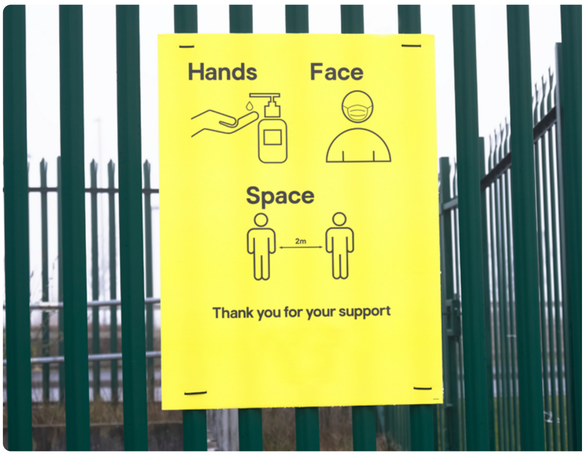

Finally, the following questions will ask how you find dealing with information around the topic of COVID-19.

51

How well informed do you feel about the COVID-19 pandemic?

- ◯ Very informed
- ◯ Good
- ◯ Satisfactory
- ◯ Poor
- ◯ Insufficiently informed

52

How do you feel about hearing/reading COVID-19 information?

- Not at all confused
- A little confused
- Quite confused
- Very confused

53

Will you or have you been vaccinated against COVID-19?

- Yes
- Likely
- Maybe
- Unlikely
- Definitely not
- Prefer not to answer

How much do you agree or disagree with the following statements?

	Strongly agree	Agree	Don't agree	Strongly disagree	Prefer not to answer
Vaccinations are important to protect myself and my family	○	○	○	○	○
Overall, I believe that vaccinations are safe.	○	○	○	○	○
Overall, I believe that vaccinations are effective.	○	○	○	○	○
Vaccination is compatible with my attitudes or religious beliefs.	○	○	○	○	○

On a scale of very easy to very difficult, how would you say it is to find information...

	Very easy	Easy	Difficult	Very difficult
About COVID-19 on the internet?	◯	◯	◯	◯
On the internet about protective behaviours that can help to prevent infection with COVID-19?	◯	◯	◯	◯
In newspapers, magazines and on TV about behaviours that can help to prevent infection with COVID-19?	◯	◯	◯	◯
About how to recognise if I am likely to be infected with COVID-19?	◯	◯	◯	◯
On how to find professional help in case of coronavirus infection	◯	◯	◯	◯
On how much i am at risk for infection with COVID-19?	◯	◯	◯	◯

On a scale of very easy to very difficult, how would you say it is to...

	Very easy	Easy	Difficult	Very Difficult
Decide how I can protect myself from COVID-19 infection based on information in the media?	○	○	○	○
Follow instructions from my doctor or pharmacist regarding how to handle the COVID-19 situation?	○	○	○	○
Use information my doctor gives me to decide how to handle a COVID-19 infection?	○	○	○	○
Use media information to decide how to handle a COVID-19 infection?	○	○	○	○
Behave in a way to avoid infecting others?	○	○	○	○

On a scale of very easy to very difficult, how would you say it is to understand...

	Very easy	Easy	Difficult	Very Difficult
Your doctor, pharmacist or nurses instructions on protective measures against COVID-19 infection?	○	○	○	○
Recommendations of authorities regarding protective measures against COVID-19 infection?	○	○	○	○
Advice from family members or friends regarding protective measures against COVID-19 infection?	○	○	○	○
Information in the media about how to protect myself against COVID-19 infection?	○	○	○	○
Risks of COVID-19 that I find on the internet?	○	○	○	○
Risks of COVID-19 that I find in				

newspapers.

58

On a scale of very easy to very difficult, how would you say it is to judge...

	Very easy	Easy	Difficult	Very Difficult
If information on COVID-19 in the media is reliable?	◯	◯	◯	◯
Which behaviours are associated with higher risk of COVID-19 infection?	◯	◯	◯	◯
What protective measures you can apply to prevent COVID-19 infection?	◯	◯	◯	◯
How much i am at risk for COVID-19 infection?	◯	◯	◯	◯
If I have been infected with COVID-19?	◯	◯	◯	◯

This content is neither created nor endorsed by Microsoft. The data you submit will be sent to the form owner.

Microsoft Forms
