# Supplementary material for: The well-being and work-related stress of senior school leaders in Wales and Northern Ireland during COVID-19 “educational leadership crisis”: A cross-sectional descriptive study
Source: PLoS One. 2024 Apr 10;19(4):e0291278. doi: 10.1371/journal.pone.0291278 (PMC11006137; doi:10.1371/journal.pone.0291278)
Supplement: S4 Fig — (DOCX) [file pone.0291278.s005.docx]

# S4 Fig Distribution of responses

## Health in general

| **How is your health in general** | **Whole sample** | **Wales** | **Northern Ireland** |
| --- | --- | --- | --- |
| *Very good* | 87 (27.1%) | 57 (33.5%) | 30 (19.9%) |
| *Good* | 139 (43.3%) | 78 (45.9%) | 61 (40.4%) |
| *Moderate* | 78 (24.1%) | 28 (16.5%) | 50 (33.1%) |
| *Bad* | 15 (5.0%) | 7 (4.1%) | 9 (6.0%) |
| *Very bad* | <5 | 0 | <5 |

| **How is your health in general** | **Males** | **Females** |
| --- | --- | --- |
| *Very good* | 31 (29.8%) | 56 (25.9%) |
| *Good* | 41 (39.4%) | 97 (44.9%) |
| *Moderate* | 27 (26.0%) | 51 (23.6%) |
| *Bad* | <5 | 12 (5.6%) |
| *Very bad* | <5 | 0 |

## WHO-5

| **WHO-5** | | **Whole sample** | **Wales** | **Northern Ireland** |
| --- | --- | --- | --- | --- |
| **Felt cheerful and in good spirits** | *None of the time* | 5 (1.6%) | <5 | <5 |
|  | *Some of the time* | 77 (24.1%) | 36 (21.2%) | 41 (27.3%) |
|  | *Less than half of the time* | 59 (18.4%) | 25 (14.7%) | 34 (22.7%) |
|  | *More than half of the time* | 65 (20.3%) | 29 (17.1%) | 36 (24.0%) |
|  | *Most of the time* | 109 (33.7%) | 77 (45.3%) | 32 (29.4%) |
|  | *All of the time* | 5 (1.6%) | <5 | <5 |
| **I have felt calm and relaxed** | *None of the time* | 31 (9.8%) | 15 (8.9%) | 16 (10.9%) |
|  | *Some of the time* | 96 (30.4%) | 43 (25.4%) | 53 (36.1%) |
|  | *Less than half of the time* | 72 (22.8%) | 36 (21.3%) | 36 (24.5%) |
|  | *More than half of the time* | 72 (22.8%) | 46 (27.2%) | 26 (17.7%) |
|  | *Most of the time* | 41 (13.0%) | 28 (16.6%) | 13 (8.8%) |
|  | *All of the time* | <5 | <5 | <5 |
| **I have felt active and vigorous** | *None of the time* | 43 (13.7%) | 16 (9.5%) | 27 (18.6%) |
|  | *Some of the time* | 77 (24.6%) | 38 (22.6%) | 39 (26.9%) |
|  | *Less than half of the time* | 91 (29.1%) | 44 (26.2%) | 47 (32.4%) |
|  | *More than half of the time* | 49 (15.7%) | 34 (20.2%) | 15 (10.3%) |
|  | *Most of the time* | 45 (14.4%) | 33 (19.6%) | 12 (8.3%) |
|  | *All of the time* | 8 (2.6%) | <5 | 5 (3.4%) |
| **I woke up feeling fresh and relaxed** | *None of the time* | 109 (34.7%) | 47 (28.0%) | 62 (42.5%) |
|  | *Some of the time* | 84 (26.8%) | 48 (28.6%) | 36 (24.7%) |
|  | *Less than half of the time* | 67 (21.3%) | 37 (22.0%) | 30 (20.5%) |
|  | *More than half of the time* | 31 (9.9%) | 20 (11.9%) | 11 (7.5%) |
|  | *Most of the time* | 21 (6.7%) | 15 (8.9%) | 6 (4.1%) |
|  | *All of the time* | <5 | <5 | <5 |
| **My daily life has been filled with things that interest me** | *None of the time* | 15 (4.8%) | 7 (4.2%) | 8 (5.6%) |
|  | *Some of the time* | 104 (33.3%) | 50 (29.8%) | 54 (37.5%) |
|  | *Less than half of the time* | 59 (18.9%) | 31 (18.5%) | 28 (19.4%) |
|  | *More than half of the time* | 61 (19.6%) | 31 (18.5%) | 30 (20.8%) |
|  | *Most of the time* | 65 (20.8%) | 44 (26.2%) | 21 (14.6%) |
|  | *All of the time* | 8 (2.5%) | 5 (3.0%) | <5 |

| **WHO-5** | | **Males** | **Females** |
| --- | --- | --- | --- |
| **Felt cheerful and in good spirits** | *None of the time* | <5 | <5 |
|  | *Some of the time* | 26 (25.0%) | 51 (23.7%) |
|  | *Less than half of the time* | 15 (14.4%) | 44 (20.5) |
|  | *More than half of the time* | 19 (18.3%) | 45 (20.9%) |
|  | *Most of the time* | 39 (37.5%) | 70 (32.6%) |
|  | *All of the time* | <5 | <5 |
| **I have felt calm and relaxed** | *None of the time* | 9 (8.7%) | 22 (10.4%) |
|  | *Some of the time* | 32 (30.8%) | 64 (30.3%) |
|  | *Less than half of the time* | 18 (17.3%) | 54 (25.6%) |
|  | *More than half of the time* | 22 (21.2%) | 49 (23.2%) |
|  | *Most of the time* | 21 (20.2%) | 20 (9.5%) |
|  | *All of the time* | <5 | <5 |
| **I have felt active and vigorous** | *None of the time* | 9 (8.7%) | 34 (16.3%) |
|  | *Some of the time* | 27 (26.2%) | 50 (23.9%) |
|  | *Less than half of the time* | 27 (26.2%) | 64 (30.6%) |
|  | *More than half of the time* | 15 (14.6%) | 33 (15.8%) |
|  | *Most of the time* | 20 (19.4%) | 25 (12.0%) |
|  | *All of the time* | 5 (4.9%) | <5 |
| **I woke up feeling fresh and relaxed** | *None of the time* | 35 (34.0%) | 74 (35.2%) |
|  | *Some of the time* | 27 (26.2%) | 57 (27.1%) |
|  | *Less than half of the time* | 20 (19.4%) | 46 (21.9%) |
|  | *More than half of the time* | 10 (9.7%) | 21 (10.0%) |
|  | *Most of the time* | 10 (9.7%) | 11 (5.2%) |
|  | *All of the time* | <5 | <5 |
| **My daily life has been filled with things that interest me** | *None of the time* | 9 (8.7%) | 6 (2.9%) |
|  | *Some of the time* | 30 (29.1%) | 74 (35.6%) |
|  | *Less than half of the time* | 20 (19.4%) | 38 (18.3%) |
|  | *More than half of the time* | 17 (16.5%) | 44 (21.2%) |
|  | *Most of the time* | 22 (33.8%) | 43 (20.7%) |
|  | *All of the time* | 5 (4.9%) | <5 |

## Sense of Coherence

| **Sense of Coherence** | **Whole sample** | **Wales** | **Northern Ireland** |
| --- | --- | --- | --- |
| Unmanageable - manageable | 3.19 ± 1.57 | 3.59 ± 1.58 | 2.73 ± 1.44 |
| Meaningless - meaningful | 4.93 ± 1.59 | 5.07 ± 1.62 | 4.78 ± 1.55 |
| Unstructured - structured | 4.38 ± 1.60 | 4.50 ± 1.6 | 4.25 ± 1.59 |
| Impossible to influence - easy to influence | 3.43 ± 1.51 | 3.70 ± 1.54 | 3.14 ± 1.41 |
| Insignificant – significant | 5.23 ± 1.70 | 5.43 ± 1.59 | 5.02 ± 1.81 |
| Unclear - clear | 4.25 ± 1.74 | 4.39 ± 1.73 | 4.09 ± 1.74 |
| Uncontrollable - controllable | 3.22 ± 1.56 | 3.36 ± 1.56 | 3.06 ± 1.56 |
| Unrewarding - rewarding | 4.32 ± 1.92 | 4.66 ± 1.93 | 3.93 ± 1.84 |
| Unpredictable - predictable | 2.64 ± 1.57 | 2.90 ± 1.60 | 2.35 ± 1.51 |

| **Sense of Coherence** | **Males** | **Females** |
| --- | --- | --- |
| Unmanageable - manageable | 3.27 ± 1.68 | 3.16 ± 1.53 |
| Meaningless - meaningful | 4.83 ± 1.62 | 5.00 ± 1.56 |
| Unstructured - structured | 4.18 ± 1.45 | 4.49 ± 1.65 |
| Impossible to influence - easy to influence | 3.39 ± 1.61 | 3.46 ± 1.45 |
| Insignificant – significant | 5.24 ± 1.66 | 5.25 ± 1.71 |
| Unclear - clear | 4.12 ± 1.70 | 4.32 ± 1.74 |
| Uncontrollable - controllable | 3.24 ± 1.49 | 3.22 ± 1.60 |
| Unrewarding - rewarding | 4.02 ± 1.98 | 4.48 ± 1.88 |
| Unpredictable - predictable | 2.79 ± 1.65 | 2.57 ± 1.54 |

## Exhaustion - BAT

| **Exhaustion – BAT** | | **Whole sample** | **Wales** | **Northern Ireland** |
| --- | --- | --- | --- | --- |
| **At work I feel mentally exhausted** | *Never* | <5 | <5 | 0 |
|  | *Rarely* | 13 (4.1%) | 12 (7.1%) | 1 (0.7%) |
|  | *Sometimes* | 86 (26.9%) | 60 (35.3%) | 26 (17.3%) |
|  | *Often* | 168 (52.5%) | 76 (44.7%) | 92 (61.3%) |
|  | *Always* | 52 (16.3%) | 21 (12.4%) | 31 (20.7%) |
| **After a day at work, I find it hard to recover my energy** | *Never* | <5 | <5 | 0 |
|  | *Rarely* | 17 (5.3%) | 12 (7.1%) | 5 (3.3%) |
|  | *Sometimes* | 64 (20.0%) | 46 (27.1%) | 18 (12.0%) |
|  | *Often* | 147 (45.9%) | 78 (45.9%) | 69 (46.0% |
|  | *Always* | 91 (28.4%) | 33 (19.4%) | 58 (38.7%) |
| **At work I feel physically exhausted** | *Never* | 6 (1.9%) | 6 (3.6%) | 0 |
|  | *Rarely* | 42 (13.3%) | 36 (21.6%) | 6 (4.0%) |
|  | *Sometimes* | 113 (35.8%) | 63 (37.7%) | 50 (33.6%) |
|  | *Often* | 110 (34.8%) | 46 (27.5%) | 64 (58.2%) |
|  | *Always* | 45 (14.2%) | 16 (9.6%) | 29 (19.5%) |

| **Exhaustion – BAT** | **Whole sample** | **Wales** | **Northern Ireland** |
| --- | --- | --- | --- |
| Low | <5 | <5 | <5 |
| Average | 32 (10.0%) | 29 (17.1%) | <5 |
| High | 122 (38.1%) | 72 (42.4%) | 50 (33.3%) |
| Very high | 163 (50.5%) | 66 (38.8%) | 97 (64.7%) |

| **Exhaustion – BAT** | | **Males** | **Females** |
| --- | --- | --- | --- |
| **At work I feel mentally exhausted** | *Never* | <5 | 0 |
|  | *Rarely* | 8 (7.7%) | 5 (2.3%) |
|  | *Sometimes* | 29 (27.9%) | 57 (26.5%) |
|  | *Often* | 53 (51.0%) | 115 (53.5%) |
|  | *Always* | 13 (12.5%) | 39 (17.7%) |
| **After a day at work, I find it hard to recover my energy** | *Never* | <5 | 0 |
|  | *Rarely* | 8 (7.7%) | 9 (4.2%) |
|  | *Sometimes* | 23 (22.1%) | 41 (19.1%) |
|  | *Often* | 46 (44.2%) | 101 (47.0%) |
|  | *Always* | 26 (25.0%) | 64 (29.8%) |
| **At work I feel physically exhausted** | *Never* | <5 | <5 |
|  | *Rarely* | 21 (20.4%) | 21 (9.9%) |
|  | *Sometimes* | 29 (28.2%) | 84 (39.6%) |
|  | *Often* | 39 (37.9%) | 71 (33.5%) |
|  | *Always* | 10 (9.7%) | 34 (16.0%) |

| **Exhaustion – BAT** | **Males** | **Females** |
| --- | --- | --- |
| Low | <5 | <5 |
| Average | 15 (14.4%) | 17 (7.9%) |
| High | 34 (32.7%) | 88 (40.9%) |
| Very high | 53 (51.0%) | 109 (50.7%) |

## Psychosomatic complaints - BAT

| **Psychosomatic complaints - BAT** | | **Whole sample** | **Wales** | **Northern Ireland** |
| --- | --- | --- | --- | --- |
| **I suffer from palpitations or chest pain** | *Never* | 129 (41.2%) | 73 (43.7%) | 56 (38.4%) |
|  | *Rarely* | 65 (20.8%) | 37 (22.2%) | 28 (19.2%) |
|  | *Sometimes* | 91 (29.1%) | 44 (26.3%) | 47 (32.2%) |
|  | *Often* | 26 (8.3%) | 12 (7.2%) | 14 (9.6%) |
|  | *Always* | <5 | <5 | <5 |
| **I suffer from stomach and/or intestinal complaints** | *Never* | 83 (26.5%) | 47 (28.1%) | 36 (24.7%) |
|  | *Rarely* | 83 (26.5%) | 52 (31.1%) | 31 (21.2%) |
|  | *Sometimes* | 91 (29.1%) | 45 (26.9%) | 46 (31.5%) |
|  | *Often* | 52 (16.6%) | 19 (11.4%) | 33 (22.6%) |
|  | *Always* | <5 | <5 | 0 (0% |
| **I often get headaches** | *Never* | 27 (8.8%) | 14 (8.6%) | 13 (9.0%) |
|  | *Rarely* | 83 (26.9%) | 53 (32.5%) | 30 (20.7%) |
|  | *Sometimes* | 90 (29.2%) | 44 (27.0%) | 46 (31.7%) |
|  | *Often* | 90 (29.2%) | 43 (26.4%) | 47 (32.5%) |
|  | *Always* | 18 (5.8%) | 9 (5.5%) | 9 (6.2%) |
| **I suffer from muscle pain e.g. neck, shoulder, back** | *Never* | 25 (8.0%) | 19 (11.4%) | 6 (4.1%) |
|  | *Rarely* | 39 (12.5%) | 28 (16.8%) | 11 (7.5%) |
|  | *Sometimes* | 98 (31.3%) | 54 (32.3%) | 44 (30.1%) |
|  | *Often* | 110 (35.1%) | 49 (29.3%) | 61 (41.8%) |
|  | *Always* | 41 (13.1%) | 17 (10.2%) | 24 (16.4%) |

| **Psychosomatic complaints - BAT** | | **Males** | **Females** |
| --- | --- | --- | --- |
| **I suffer from palpitations or chest pain** | *Never* | 50 (49.0%) | 79 (37.6%) |
|  | *Rarely* | 23 (22.5%) | 42 (20.0%) |
|  | *Sometimes* | 22 (21.6%) | 68 (32.4%) |
|  | *Often* | 7 (6.9%) | 19 (9.0%) |
|  | *Always* | 0 | <5 |
| **I suffer from stomach and/or intestinal complaints** | *Never* | 30 (29.4%) | 53 (25.2%) |
|  | *Rarely* | 36 (35.3%) | 47 (22.4%) |
|  | *Sometimes* | 27 (26.5%) | 63 (30.0%) |
|  | *Often* | 9 (8.8%) | 43 (20.5%) |
|  | *Always* | 0 | <5 |
| **I often get headaches** | *Never* | 16 (15.8%) | 11 (5.3%) |
|  | *Rarely* | 34 (33.7%) | 49 (23.8%) |
|  | *Sometimes* | 31 (30.7%) | 58 (28.2%) |
|  | *Often* | 16 (15.8%) | 74 (35.9%) |
|  | *Always* | <5 | 14 (6.8%) |
| **I suffer from muscle pain e.g. neck, shoulder, back** | *Never* | 18 (17.5%) | 7 (3.3%) |
|  | *Rarely* | 16 (15.5%) | 23 (11.0%) |
|  | *Sometimes* | 35 (34.0%) | 63 (30.1%) |
|  | *Often* | 26 (25.2%) | 83 (39.7%) |
|  | *Always* | 8 (7.8%) | 33 (15.8%) |

| **Psychosomatic complaints – BAT** | **Males** | **Females** |
| --- | --- | --- |
| Low | 38 (38.4%) | 42 (21.1%) |
| Average | 45 (45.5%) | 75 (37.7%) |
| High | 13 (13.1%) | 69 (34.7%) |
| Very high | <5 | 13 (6.5%) |

## Self-endangering behaviour

**Extensification of work**

| **Self-endangering behaviour – extensification of**  **work**  **In the last 3 months…** | | **Whole sample** | **Wales** | **Northern Ireland** |
| --- | --- | --- | --- | --- |
| **Been available for your colleagues, pupils and parents in your free time?** | *Never* | 6 (1.9%) | <5 | 5 (3.3%) |
|  | *Almost never* | 30 (9.4%) | 14 (8.3%) | 16 (10.6%) |
|  | *Sometimes* | 65 (20.3%) | 25 (14.8%) | 40 (26.5%) |
|  | *Fairly often* | 90 (28.1%) | 60 (35.5%) | 30 (19.9%) |
|  | *Very often* | 129 (40.3%) | 69 (40.8%) | 60 (39.7%) |
| **Given up leisure activities in favour of work?** | *Never* | <5 | <5 | <5 |
|  | *Almost never* | 11 (3.4%) | 6 (3.6%) | 5 (3.3%) |
|  | *Sometimes* | 68 (21.3%) | 40 (23.7%) | 28 (18.5%) |
|  | *Fairly often* | 100 (31.3%) | 56 (33.1%) | 44 (29.1%) |
|  | *Very often* | 139 (43.4%) | 66 (39.1%) | 73 (48.3%) |
| **Forgone getting sufficient sleep in favour of work?** | *Never* | 7 (2.2%) | 5 (3.0%) | <5 |
|  | *Almost never* | 33 (10.3%) | 19 (11.2%) | 14 (9.3%) |
|  | *Sometimes* | 77 (24.1%) | 47 (27.8%) | 30 (20.0%) |
|  | *Fairly often* | 93 (29.2%) | 48 (28.4%) | 45 (30.0%) |
|  | *Very often* | 109 (34.2%) | 50 (29.6%) | 59 (39.3%) |
| **Worked extra hours in your free time (after work, during holidays, at weekends, on public holidays)?** | *Never* | <5 | <5 | <5 |
|  | *Almost never* | <5 | <5 | <5 |
|  | *Sometimes* | 23 (7.2%) | 10 (5.9%) | 13 (8.6%) |
|  | *Fairly often* | 66 (20.6%) | 38 (22.5%) | 28 (18.5%) |
|  | *Very often* | 227 (70.9%) | 120 (71.0%) | 107 (70.9%) |
| **Waived breaks (short or lunch break) during your working hours?** | *Never* | <5 | <5 | <5 |
|  | *Almost never* | <5 | <5 | <5 |
|  | *Sometimes* | 11 (3.4%) | 5 (3.0%) | 6 (4.0%) |
|  | *Fairly often* | 42 (13.1%) | 20 (11.8%) | 22 (14.6%) |
|  | *Very often* | 263 (82.2%) | 142 (84.0%) | 121 (80.1%) |
| **Worked longer than contractually agreed?** | *Never* | <5 | <5 | <5 |
|  | *Almost never* | <5 | <5 | 0 |
|  | *Sometimes* | 16 (5.0%) | 10 (6.0%) | 6 (4.0%) |
|  | *Fairly often* | 30 (9.5%) | 18 (10.8%) | 12 (8.0%) |
|  | *Very often* | 270 (85.2%) | 138 (82.6%) | 132 (88.0%) |

| **Self-endangering behaviour – extensification of**  **work**  **In the last 3 months…** | | **Males** | **Females** |
| --- | --- | --- | --- |
| **Been available for your colleagues, pupils and parents in your free time?** | *Never* | 5 (4.9%) | <5 |
|  | *Almost never* | 8 (7.8%) | 22 (10.2%) |
|  | *Sometimes* | 19 (18.4%) | 46 (21.3%) |
|  | *Fairly often* | 37 (35.9%) | 53 (24.5%) |
|  | *Very often* | 34 (33.0%) | 94 (43.5%) |
| **Given up leisure activities in favour of work?** | *Never* | <5 | <5 |
|  | *Almost never* | 5 (4.9%) | 6 (2.8%) |
|  | *Sometimes* | 27 (26.2%) | 41 (19.0%) |
|  | *Fairly often* | 38 (36.9%) | 62 (28.7%) |
|  | *Very often* | 32 (31.1%) | 106 (49.1%) |
| **Forgone getting sufficient sleep in favour of work?** | *Never* | <5 | <5 |
|  | *Almost never* | 15 (14.6%) | 18 (8.4%) |
|  | *Sometimes* | 27 (26.2%) | 50 (23.3%) |
|  | *Fairly often* | 29 (28.2%) | 64 (29.8%) |
|  | *Very often* | 29 (28.2%) | 79 (36.7%) |
| **Worked extra hours in your free time (after work, during holidays, at weekends, on public holidays)?** | *Never* | 0 | 0 |
|  | *Almost never* | <5 | <5 |
|  | *Sometimes* | 13 (12.6%) | 10 (4.6%) |
|  | *Fairly often* | 20 (19.4%) | 46 (21.3%) |
|  | *Very often* | 68 (66.0%) | 158 (73.1%) |
| **Waived breaks (short or lunch break) during your working hours?** | *Never* | <5 | <5 |
|  | *Almost never* | 0 | <5 |
|  | *Sometimes* | 7 (6.8%) | <5 |
|  | *Fairly often* | 11 (10.7%) | 31 (14.4%) |
|  | *Very often* | 83 (80.6%) | 179 (82.9%) |
| **Worked longer than contractually agreed?** | *Never* | 0 | 0 |
|  | *Almost never* | <5 | 0 |
|  | *Sometimes* | 11 (10.8%) | 5 (2.3%) |
|  | *Fairly often* | 9 (8.8%) | 21 (9.8%) |
|  | *Very often* | 81 (79.4%) | 188 (87.9%) |

### Intensification of work

| **Self-endangering behaviour – intensification of**  **work**  **In the last 3 months how often have you worked at a pace that…** | | **Whole sample** | **Wales** | **Northern Ireland** |
| --- | --- | --- | --- | --- |
| **You find burdensome** | *Never* | <5 | <5 | <5 |
|  | *Almost never* | 9 (2.8%) | 8 (4.7%) | <5 |
|  | *Sometimes* | 69 (21.5%) | 48 (28.2%) | 21 (13.9%) |
|  | *Fairly often* | 133 (41.4%) | 67 (39.4%) | 66 (43.7%) |
|  | *Very often* | 106 (33.0%) | 44 (25.9%) | 62 (41.1%) |
| **You cannot sustain in the long term** | *Never* | <5 | <5 | <5 |
|  | *Almost never* | 13 (4.0%) | 12 (7.1%) | <5 |
|  | *Sometimes* | 45 (14.0%) | 32 (18.8%) | 13 (8.6%) |
|  | *Fairly often* | 103 (32.1%) | 57 (33.5%) | 46 (30.5%) |
|  | *Very often* | 158 (49.2%) | 68 (40.0%) | 90 (59.6%) |
| **You know is not good for you** | *Never* | <5 | <5 | <5 |
|  | *Almost never* | 7 (2.2%) | 6 (3.5%) | <5 |
|  | *Sometimes* | 45 (14.0%) | 34 (20.0%) | 11 (7.3%) |
|  | *Fairly often* | 92 (28.7%) | 53 (31.2%) | 39 (25.8%) |
|  | *Very often* | 174 (54.2%) | 75 (44.1%) | 99 (65.6%) |

| **Self-endangering behaviour – intensification of**  **work**  **In the last 3 months how often have you worked at a pace that…** | | **Males** | **Females** |
| --- | --- | --- | --- |
| **You find burdensome** | *Never* | <5 | <5 |
|  | *Almost never* | 5 (4.8%) | <5 |
|  | *Sometimes* | 21 (20.2%) | 48 (22.2%) |
|  | *Fairly often* | 46 (44.2%) | 87 (40.3%) |
|  | *Very often* | 30 (28.8%) | 75 (34.7%) |
| **You cannot sustain in the long term** | *Never* | 0 | <5 |
|  | *Almost never* | 7 (6.7%) | 6 (2.8%) |
|  | *Sometimes* | 16 (15.4%) | 29 (13.4%) |
|  | *Fairly often* | 34 (32.7%) | 69 (31.9%) |
|  | *Very often* | 47 (45.2%) | 110 (50.9%) |
| **You know is not good for you** | *Never* | <5 | <5 |
|  | *Almost never* | <5 | <5 |
|  | *Sometimes* | 22 (21.2%) | 23 (10.6%) |
|  | *Fairly often* | 29 (27.9%) | 63 (29.2%) |
|  | *Very often* | 49 (47.1%) | 124 (57.4%) |

### Quality reduction

| **Self-endangering behaviour – quality reduction**  **In the last 3 months how often have you been required to…** | | **Whole sample** | **Wales** | **Northern Ireland** |
| --- | --- | --- | --- | --- |
| **Be satisfied with a lower quality of work then your normally would be?** | *Never* | 15 (4.7%) | 11 (6.5%) | <5 |
|  | *Almost never* | 42 (13.1%) | 31 (18.3%) | 11 (7.3%) |
|  | *Sometimes* | 144 (48.4%) | 78 (46.2%) | 77 (51.0%) |
|  | *Fairly often* | 77 (24.1%) | 40 (23.7%) | 37 (24.5%) |
|  | *Very often* | 31 (9.7%) | 9 (5.3%) | 22 (14.6%) |
| **Lower your own work output standard?** | *Never* | 22 (6.9%) | 14 (8.3%) | 8 (5.3%) |
|  | *Almost never* | 73 (22.9%) | 46 (27.4%) | 27 (17.9%) |
|  | *Sometimes* | 141 (44.2%) | 72 (42.9%) | 69 (45.7%) |
|  | *Fairly often* | 60 (18.8%) | 32 (19.0%) | 28 (18.5%) |
|  | *Very often* | 23 (7.2%) | <5 | 19 (12.6%) |
| **Carry out more superficial work (e.g. clerical tasks, admin)?** | *Never* | <5 | 0 | <5 |
|  | *Almost never* | 12 (3.8%) | 9 (5.3%) | <5 |
|  | *Sometimes* | 47 (14.7%) | 30 (17.8%) | 17 (11.3) |
|  | *Fairly often* | 111 (34.8%) | 67 (39.6%) | 44 (29.3%) |
|  | *Very often* | 148 (46.4%) | 63 (37.3%) | 85 (57.4%) |

| **Self-endangering behaviour – quality reduction**  **In the last 3 months how often have you been required to…** | | **Males** | **Females** |
| --- | --- | --- | --- |
| **Be satisfied with a lower quality of work then your normally would be?** | *Never* | 6 (5.8%) | 9 (4.2%) |
|  | *Almost never* | 11 (10.7%) | 31 (14.4%) |
|  | *Sometimes* | 47 (45.6%) | 108 (50.0%) |
|  | *Fairly often* | 28 (27.2%) | 48 (22.2%) |
|  | *Very often* | 11 (10.7%) | 20 (9.3%) |
| **Lower your own work output standard?** | *Never* | 8 (7.8%) | 14 (6.5%) |
|  | *Almost never* | 22 (21.4%) | 51 (23.7%) |
|  | *Sometimes* | 47 (45.6%) | 94 (43.7%) |
|  | *Fairly often* | 18 (17.5%) | 41 (19.1%) |
|  | *Very often* | 8 (7.8%) | 15 (7.0%) |
| **Carry out more superficial work (e.g. clerical tasks, admin)?** | *Never* | <5 | 0 |
|  | *Almost never* | 5 (4.9%) | 7 (3.2%) |
|  | *Sometimes* | 17 (16.7%) | 30 (13.9%) |
|  | *Fairly often* | 40 (39.2%) | 70 (32.4%) |
|  | *Very often* | 39 (38.2%) | 109 (50.5%) |

## Perceived stress

| **Perceived stress**  **In the last month how often have you…** | | **Whole sample** | **Wales** | **Northern Ireland** |
| --- | --- | --- | --- | --- |
|  | | | | |
| **Been upset because of something that happened unexpectedly?** | *Never* | 15 (4.7%) | 15 (8.8%) | 0 |
|  | *Almost never* | 59 (18.4%) | 44 (25.9%) | 15 (9.9%) |
|  | *Sometimes* | 154 (48.0%) | 73 (42.9%) | 81 (53.6%) |
|  | *Fairly often* | 71 (22.1%) | 29 (17.1%) | 42 (27.8%) |
|  | *Very often* | 22 (6.9%) | 9 (5.3%) | 13 (8.6%) |
| **Felt that you were unable to control the important things?** | *Never* | 12 (3.8%) | 11 (6.5%) | 1 (0.7%) |
|  | *Almost never* | 44 (13.8%) | 29 (17.1%) | 15 (10.0%) |
|  | *Sometimes* | 132 (41.3%) | 66 (38.8%) | 66 (44.0%) |
|  | *Fairly often* | 93 (29.1%) | 47 (27.6%) | 46 (30.7%) |
|  | *Very often* | 39 (12.2%) | 17 (10.0%) | 22 (30.7%) |
| **Felt nervous and "stressed" at school?** | *Never* | 9 (2.8%) | 8 (4.7%) | 1 (0.7%) |
|  | *Almost never* | 45 (14.2%) | 36 (21.3%) | 9 (6.1%) |
|  | *Sometimes* | 101 (31.9%) | 52 (30.8%) | 49 (33.1%) |
|  | *Fairly often* | 103 (32.5%) | 48 (28.4%) | 55 (37.2%) |
|  | *Very often* | 59 (18.6%) | 25 (14.8%) | 34 (23.0%) |
| **Found that you could not cope with all your work tasks?** | *Never* | 10 (3.1%) | 10 (5.9%) | 0 ( |
|  | *Almost never* | 62 (19.4%) | 47 (27.6%) | 15 (10.0%) |
|  | *Sometimes* | 132 (41.3%) | 57 (33.5%) | 75 (50.0%) |
|  | *Fairly often* | 87 (26.9%) | 40 (23.5%) | 47 (31.3%) |
|  | *Very often* | 29 (9.1%) | 16 (9.4%) | 13 (8.7%) |
| **Been angered because of things that were outside of your control at work?** | *Never* | 10 (3.1%) | 9 (5.3%) | <5 |
|  | *Almost never* | 61 (19.0%) | 37 (21.8%) | 24 (15.9%) |
|  | *Sometimes* | 105 (32.7%) | 60 (35.3%) | 45 (29.8%) |
|  | *Fairly often* | 97 (30.2%) | 40 (23.5%) | 57 (37.7%) |
|  | *Very often* | 48 (15.0%) | 24 (14.1%) | 24 (15.9%) |
| **Felt difficulties at work were piling up so high that you could not deal with them?** | *Never* | 24 (7.5%) | 16 (9.5%) | 8 (5.3%) |
|  | *Almost never* | 89 (27.9%) | 48 (28.4%) | 41 (27.3%) |
|  | *Sometimes* | 151 (47.3%) | 69 (40.8%) | 82 (54.7%) |
|  | *Fairly often* | 45 (14.1%) | 27 (16.0%) | 18 (12.0%) |
|  | *Very often* | 10 (3.1%) | 9 (5.3%) | <5 |
|  | | | | |
| **Felt confident about your ability to handle your professional work- related problems caused by the COVID- 19 pandemic?** | *Never* | 6 (1.9%) | 2 (1.2%) | <5 |
|  | *Almost never* | 31 (9.7%) | 13 (7.6%) | 18 (11.9%) |
|  | *Sometimes* | 124 (38.6%) | 56 (32.9%) | 68 (45.0%) |
|  | *Fairly often* | 122 (38.0%) | 69 (40.6%) | 53 (35.1%) |
|  | *Very often* | 38 (11.8%) | 30 (17.6%) | 8 (5.3%) |
| **How often have you felt that things at your work at school were going your way** | *Never* | <5 | 0 | <5 |
|  | *Almost never* | 31 (9.7%) | 19 (61.3%) | 12 (8.0%) |
|  | *Sometimes* | 120 (37.6%) | 66 (39.1%) | 54 (36.0%) |
|  | *Fairly often* | 116 (36.4%) | 73 (43.2%) | 43 (28.7%) |
|  | *Very often* | 48 (15.0%) | 11 (6.5%) | 37 (24.7%) |
| **Been able to control irritations at your work?** | *Never* | 30 (9.4%) | <5 | 29 (19.5%) |
|  | *Almost never* | 74 (23.2%) | 18 (10.6%) | 56 (37.6%) |
|  | *Sometimes* | 98 (30.7%) | 54 (31.8%) | 44 (29.5%) |
|  | *Fairly often* | 82 (25.7%) | 65 (38.2%) | 17 (11.4%) |
|  | *Very often* | 35 (11.0%) | 32 (18.8%) | <5 |
| **Felt that you were on top of things at work?** | *Never* | 12 (3.8%) | 10 (5.9%) | <5 |
|  | *Almost never* | 61 (19.1%) | 50 (29.6%) | 11 (7.3%) |
|  | *Sometimes* | 113 (35.4%) | 63 (37.3%) | 50 (33.3%) |
|  | *Fairly often* | 106 (33.2%) | 42 (24.9%) | 64 (42.7%) |
|  | *Very often* | 27 (8.5%) | <5 | 23 (15.3%) |

| **Perceived stress**  **In the last month how often have you…** | | **Males** | **Females** |
| --- | --- | --- | --- |
| **Perceived helplessness** | | | |
| **Been upset because of something that happened unexpectedly?** | *Never* | 9 (8.7%) | 6 (2.8%) |
|  | *Almost never* | 27 (26.0%) | 32 (14.8%) |
|  | *Sometimes* | 38 (36.5%) | 116 (53.7%) |
|  | *Fairly often* | 24 (23.1%) | 47 (21.8%) |
|  | *Very often* | 6 (5.8%) | 15 (6.9%) |
| **Felt that you were unable to control the important things?** | *Never* | 6 (5.8%) | 6 (2.8%) |
|  | *Almost never* | 14 (13.5%) | 30 (14.0%) |
|  | *Sometimes* | 47 (45.2%) | 85 (39.5%) |
|  | *Fairly often* | 27 (26.0%) | 66 (30.7%) |
|  | *Very often* | 10 (9.6%) | 28 (13.0%) |
| **Felt nervous and "stressed" at school?** | *Never* | 5 (4.9%) | <5 |
|  | *Almost never* | 17 (16.7%) | 28 (13.1%) |
|  | *Sometimes* | 28 (27.5%) | 73 (34.1%) |
|  | *Fairly often* | 37 (36.3%) | 66 (30.8%) |
|  | *Very often* | 15 (14.7%) | 43 (20.1%) |
| **Found that you could not cope with all your work tasks?** | *Never* | 6 (5.8%) | <5 |
|  | *Almost never* | 24 (32.1%) | 38 (17.7%) |
|  | *Sometimes* | 44 (42.3%) | 88 (40.9%) |
|  | *Fairly often* | 23 (22.1%) | 64 (39.8%) |
|  | *Very often* | 7 (6.7%) | 21 (9.8%) |
| **Been angered because of things that were outside of your control at work?** | *Never* | <5 | 6 (2.8%) |
|  | *Almost never* | 25 (24.0%) | 36 (16.7%) |
|  | *Sometimes* | 30 (28.8%) | 74 (34.3%) |
|  | *Fairly often* | 28 (26.9%) | 69 (31.9%) |
|  | *Very often* | 17 (16.3%) | 31 (14.4%) |
| **Felt difficulties at work were piling up so high that you could not deal with them?** | *Never* | 11 (10.6%) | 13 (6.1%) |
|  | *Almost never* | 27 (26.0%) | 62 (29.0%) |
|  | *Sometimes* | 47 (45.2%) | 104 (48.6%) |
|  | *Fairly often* | 15 (14.4%) | 29 (13.6%) |
|  | *Very often* | <5 | 6 (2.8%) |
| **Perceived self-efficacy** | | | |
| **Felt confident about your ability to handle your professional work- related problems caused by the COVID- 19 pandemic?** | *Never* | <5 | <5 |
|  | *Almost never* | 16 (15.4%) | 15 (6.9%) |
|  | *Sometimes* | 43 (41.3%) | 81 (37.5%) |
|  | *Fairly often* | 27 (26.0%) | 95 (44.0%) |
|  | *Very often* | 16 (15.4%) | 21 (9.7%) |
| **How often have you felt that things at your work at school were going your way** | *Never* | <5 | <5 |
|  | *Almost never* | 16 (15.4%) | 15 (7.0%) |
|  | *Sometimes* | 37 (35.6%) | 83 (38.8%) |
|  | *Fairly often* | 39 (37.5%) | 77 (36.0%) |
|  | *Very often* | 11 (10.6%) | 36 (16.8%) |
| **Been able to control irritations at your work?** | *Never* | 6 (5.8%) | 24 (11.2%) |
|  | *Almost never* | 22 (21.4%) | 52 (24.2%) |
|  | *Sometimes* | 32 (31.1%) | 66 (30.7%) |
|  | *Fairly often* | 29 (28.2%) | 53 (24.7%) |
|  | *Very often* | 14 (13.6%) | 20 (9.3%) |
| **Felt that you were on top of things at work?** | *Never* | <5 | 7 (3.3%) |
|  | *Almost never* | 16 (15.5%) | 45 (20.9%) |
|  | *Sometimes* | 37 (35.9%) | 76 (35.3%) |
|  | *Fairly often* | 39 (37.9%) | 67 (31.2%) |
|  | *Very often* | 7 (6.8%) | 20 (9.3%) |
