# Supplementary material for: The well-being and work-related stress of senior school leaders in Wales and Northern Ireland during COVID-19 “educational leadership crisis”: A cross-sectional descriptive study
Source: PLoS One. 2024 Apr 10;19(4):e0291278. doi: 10.1371/journal.pone.0291278 (PMC11006137; doi:10.1371/journal.pone.0291278)
Supplement: Attachment — Submitted filename: PLOS ONE Review.docx [file pone.0291278.s006.docx]

Overall, I thought this was a well-written paper. Most of my feedback is rather minor. The major issue I believe worth highlighting is the need for some sort of conceptual/theoretical framing section. I think rooting this study within crises, leadership, and what research has come out about COVID-19 in education is needed (presently, not enough has been inserted). Otherwise, most of the paper is strong and the claims are supported. Please see comments below.

Intro

Page 9, line 25: this sentence read a little awkward. I recommend revising and shortening.

Page 9, line 29: I wouldn’t say it impacted everyone, but rather most people. Some contexts didn’t change a thing.

Page 11, line 74: how many people were in the convenience sample, for both Whales and Northern Ireland.

General: After the intro, some sort of conceptual framing or theory section is needed to illuminate your findings. Something around crisis leadership and management would be useful. Also, expand on what current COVID-19 research in education has found. This is a key piece missing right now. Tie these back to the various concepts the survey studied. Theory helps to bring clarity.

Methods

General: I think a paragraph about the contexts of Wales and Northern Ireland in response to the pandemic is needed. This is your context, so help the reader understand it a little more.

Page 12: say how many questions the survey had in the actual text, and give some key examples. Help the reader better understand its scope in the text. Also, there are various categories in the survey you discuss. First, upfront, say each of the categories that you use (as a list), then expand upon them later (as you do). This helps the reader follow your organization of information. Also, about how long did it take to complete the full survey? Again, just give a little more detail upfront. More is better here.

Data analysis: This section seemed far too short. Give a little more color to it. Two sentences is not enough.

Results

General: It seems like the health and well-being data contradicted each other (to a degree). Thus, some discussion of this is required in the discussion section. I would have assumed lower health given the other subsection scores.

General: Do you have more data on the retention rates of senior leaders in Wales and Northern Ireland? If so, that would reinforce the claims you make by a lot. Tying to actual burnout levels now would be great.

Page 16: Before breaking down each category, I recommend some broad, interesting findings in a short paragraph. Give the reader a taste, then expand upon those findings in each subsection (as you do). Remember, show the forest, then the trees! This is common practice.
