# Supplementary material for: The well-being and work-related stress of senior school leaders in Wales and Northern Ireland during COVID-19 “educational leadership crisis”: A cross-sectional descriptive study
Source: PLoS One. 2024 Apr 10;19(4):e0291278. doi: 10.1371/journal.pone.0291278 (PMC11006137; doi:10.1371/journal.pone.0291278)
Supplement: Attachment — Submitted filename: Response to reviewers.docx [file pone.0291278.s007.docx]

**Response to reviewer**

Firstly, on behalf of the co-authors, we would like to express our sincere gratitude to the reviewer for their productive and positive comments regarding the manuscript. This review process has significantly improved the manuscript, and we are grateful for the time taken to provide such useful feedback.

**Introduction**

**Page 9, line 25: this sentence read a little awkward. I recommend revising and shortening.**

Thank you for drawing our attention to this, we have reworded the first five lines of the introduction to improve clarity of expression:

Page 3 line 24-29:

*The COVID-19 pandemic caused by the transmission of severe acute respiratory syndrome coronavirus 2 (SARS-CoV-2) resulted in unprecedented societal changes. In response, a range of public health measures were implemented to reduce social contacts and transmission of SARS-CoV-2. This included changes to the delivery of learning, teaching and assessment within educational settings and contexts. Measures included full or partial closure of face-to-face provision, a move to hybrid and blended learning, and the introduction of a variety of school-based measures upon the full return to education [1–6].*

**Page 9, line 29: I wouldn’t say it impacted everyone, but rather most people. Some contexts didn’t change a thing.**

We have addressed this by rewording from *all people*, to all *layers of education*, thus, whilst not all of the education population were impacted, there were impacts at each layer from pupils through to leadership teams.

Page 3 line 30:

*These significant and prolonged changes to the delivery of teaching and learning impacted all layers of education.*

**Page 11, line 74: how many people were in the convenience sample, for both Whales and Northern Ireland.**

We have now updated the manuscript with sample information:

Page 7 line 192-193:

*A convenience sample of participants (total: n=323, Wales: n=172, Northern Ireland: n=151) were recruited via email, social media and through education stakeholders.*

**General: After the intro, some sort of conceptual framing or theory section is needed to illuminate your findings. Something around crisis leadership and management would be useful. Also, expand on what current COVID-19 research in education has found. This is a key piece missing right now. Tie these back to the various concepts the survey studied. Theory helps to bring clarity.**

Thank you for highlighting this important conceptual framework suggestion. We agree with your points and believe more direct framing of the study through the theoretical lens of educational crisis leadership would significantly strengthen the manuscript.

We have incorporated more explicit discussions of educational crisis leadership conceptual frameworks throughout the manuscript, including the Introduction, Methods and Materials, Discussion and Conclusion, please find below details of these revisions:

Title

Page 1:

*The well-being and work-related stress of senior school leaders in Wales and Northern Ireland during COVID-19 “educational leadership crisis”: A cross-sectional descriptive study*

Page 2 line 5-6:

leaders were exposed to high demands relating to the numerous challenges they had to manage during a “*crisis leadership*” period.

Page 3 line 40-42:

Senior school leaders are responsible for all aspects of school life and therefore had to cope with high demands due to the COVID-19 pandemic, a period defined as crisis leadership [10,11].

Page 3-4 line 43-79:

*The term crisis leadership is characterised as an urgent situation requiring immediate and decisive action by an organisation or leaders of the organisation [12]. In the context of education, evidence regarding crisis leadership is limited but explores how educational leaders manage a period of “unexpected, fundamental disruption to school functioning with potentially high consequences for the organization, its stakeholders, and its reputation” [13, p.315]. To date, scoping reviews demonstrate the majority of research focuses on the response to and recovery from natural disasters and human-made crises, with a significant lack of research on educational leadership during public health crises [14]. However, crisis leadership in education was brought to the forefront during COVID-19, and a growing body of evidence has drawn attention to its importance in securing stability for learners, parents, staff and the community during a time of unprecedented disruption [9,12,13,15]. Recent work has focussed on developing frameworks to understand the processes behind the management of educational crises, such as the five phase cycle of mitigation/prevention, preparedness, response, recovery and learning [13]. Whilst our understanding of the conditions that enable effective educational crisis leadership is growing [13,14], these frameworks focus on the skills and attributes of leaders during the process of crisis leadership, neglecting outcomes such as how school leaders deal with and are impacted by this period. Thus, there is a gap in empirical research examining the impact on how school leaders experienced crisis leadership during COVID-19. This is important because factors such as well-being and work-related stress may be impacted which are fundamental during and after periods of crisis leadership.*

Page 5 line 141-143:

*This study aimed to examine the working situation, well-being and work-related stress of senior school leaders in Wales and Northern Ireland and explore gender differences as a whole sample in a period of educational crisis leadership during the COVID-19 pandemic 2021 and 2022.*

Page 7 line 178-186:

*Conceptual framework*

*This study is conceptually positioned within the framing of educational crisis leadership. Defined above, this study recognises this as a period of “unexpected, fundamental disruption to school functioning with potentially high consequences for the organization, its stakeholders, and its reputation” [13, p.315] that requires immediate and decisive action by school leaders [12]. Whilst this study does not aim to examine the conditions and factors associated with effective educational crisis leadership, it aims to examine how school leaders experienced crisis leadership and the perceived impact this had on their working situation, well-being and work-related stress. These are currently neglected areas of study with the literature. In recognition of current frameworks on educational crisis leadership [13], this study informs the recovery and learning phases of these models based on school leaders’ experiences.*

Page 19 line 443-454:

*During the COVID-19 pandemic, senior school leaders were exposed to a period of “crisis leadership” [10]. Whilst evidence has focussed on developing frameworks to better understand how school leaders can manage and navigate periods of crisis leadership, there is a dearth of evidence examining the impacts this can have and how they experience this unprecedented disruption. Findings from this study contribute to the limited knowledge base understanding the impact this period of crisis leadership had on specific aspects of school leaders’ physical and mental health. This is important in order to enable the education and health systems to respond with a targeted approach and appropriate resources, and in addressing the recovery and learning phases of educational crisis leadership models based on the experience of school leaders [13]. This evidence base can shape and inform policy and practice and directly address the working situation and well-being experiences of leaders in the education system. This should be considered fundamental components embedded within frameworks of educational crisis leadership and future crises. The findings in the current study supports other work in this area both nationally and internationally [20,33–37,59].*

Page 24-25 line 587-597:

*This study expands current knowledge on the impacts of the COVID-19 pandemic and crisis leadership on educational leaders in Wales and Northern Ireland. Applying the findings through the lens of educational crisis leadership frameworks, it is essential to consider the last phases of recovery and learning to minimise and address impacts and increase preparedness [13].*

**Methods**

**General: I think a paragraph about the contexts of Wales and Northern Ireland in response to the pandemic is needed. This is your context, so help the reader understand it a little more.**

We agree that adding both general and Covid-related context to Wales and Northern Ireland would be useful for the reader. To address your suggestion, we have included an additional sub-section in this chapter:

Page 6 line 153-168:

*Context*

*Education and health are devolved responsibilities of the respective governments of Wales and Northern Ireland within the UK. There was some general consistency in both countries’ educational response to COVID-19 including periods of closure and the reopening of face-to-face educational provision, blended and hybrid learning, and the implementation of school-based measures (e.g. social distancing) [38,39]. However, there was variation in the timings of implementation and relaxation of measures during the entirety of the COVID-19 pandemic and within the study period [38,39].*

*In Wales, the collection of data shortly followed the re-opening of face-to-face education (February-April 2021), and encompassed requirements of self-isolation and hybrid learning (June and July 2021), teacher-based assessments (June 2021), increases in COVID-19 cases linked to schools (July and October 2021), regular lateral flow testing of pupils (secondary) and teachers (September 2021) and the reintroduction of school-based public health measures (wearing face coverings). In addition to this, schools across Wales were preparing to roll out a new curriculum, the Curriculum for Wales [40] following significant education reform [41]. In Northern Ireland, the data collection period was later into the pandemic. Whilst education had returned somewhat to pre-pandemic delivery, this was amongst a backdrop of continued high COVID-19 rates, significant staff and pupil absences (January and February 2022), and ongoing COVID-related impacts and sustained pressures on the education system [39].*

We have also further addressed this as a limitation of the study:

Page 26 line 661-663:

*Both countries implemented various public measures at different timepoints, including the closure and reopening of face-to-face education settings.*

**Page 12: say how many questions the survey had in the actual text, and give some key examples. Help the reader better understand its scope in the text. Also, there are various categories in the survey you discuss. First, upfront, say each of the categories that you use (as a list), then expand upon them later (as you do). This helps the reader follow your organization of information. Also, about how long did it take to complete the full survey? Again, just give a little more detail upfront. More is better here.**

To address this, we have provided information in-text about categories and sub-categories (see below) and an additional supplementary file (S1 Fig) that contains a full breakdown of all categories and sub-categories of the full survey.

Page 8 line 213-226:

*The COVID-19 School Principal Survey [32] was designed by the international COVID-HL network and contained 33 questions. The original survey consisted of various categories and sub-categories; socio-demographic information (e.g. gender, type of school), current work situation (e.g. Sense of Coherence, perceived stress), health information in the context of COVID-19 (e.g. health literacy), health promotion and prevention in school (e.g. health promoting school factors) and health situation (general health, well-being). A full breakdown of categories and sub-categories is presented in S1 Fig. For the purpose of this study, questions relating to work situation, health, well-being, work-related Sense of Coherence, exhaustion and psychosomatic complaints, self-endangering behaviour and perceived stress were used for analyses. The survey was adapted for Wales and Northern Ireland including country specific wording, additional questions seeking demographic characteristics, and minor re-structuring (S2 and S3 Fig). The inclusion criteria for the survey were any senior leadership staff (headteacher, deputy headteacher, senior leadership team). The survey was completed on Microsoft Forms and took an average completion time of 30 minutes for participants in Wales and 28 minutes for participants in Northern Ireland. A full copy of the survey for Wales and Northern Ireland is available in the S2 and S3 Fig. A full copy of the original survey is available upon request.*

For the purpose of providing this contextual information for the reader we feel this is best placed in the supplementary information to avoid large word limits. We have also included an asterisk to denounce sub-categories of questions used within analyses. As categories and sub-categories, full surveys for Wales and Northern Ireland are available in the supplementary information, and individual question and response examples are provided in the Methods and materials section, we feel this sufficiently provides the information the reader requires.

We have added in average completion time of the survey:

Page 8 line 223-224:

*The survey was completed on Microsoft Forms and took an average completion time of 30 minutes for participants in Wales and 28 minutes for participants in Northern Ireland*

**Data analysis: This section seemed far too short. Give a little more color to it. Two sentences is not enough.**

Thank you for highlighting this, we have included additional information about the data analysis process:

Page 12 line 324-330:
*Raw data were downloaded from Microsoft Forms, cleaned and checked for duplicates, and unique participant ID numbers generated. Data were then handled using IBM SPSS Statistics (version 28.0 for Mac). Raw survey responses were coded following COVID-HL study documentation protocol, this ensures consistency across international study data. Next, descriptive statistics were calculated describing frequencies and percentages, mean values and standard deviations for the variables listed above. This was done in three phases, as a whole sample, by country and by gender. Independent samples t-tests explored differences between gender, with the level of statistical significance set as a two-sided p < 0.05.*

**Results**

**General: It seems like the health and well-being data contradicted each other (to a degree). Thus, some discussion of this is required in the discussion section. I would have assumed lower health given the other subsection scores.**

Thank you for this observation, we agree there is some contradiction regarding the reporting of general health compared to other measures of well-being and stress. This is an interesting finding as it is fair to assume this would be lower given the low well-being, high work-related stress amongst others experienced by senior leaders in this study. We have discussed this finding in relation to wider longitudinal studies, suggesting possible protective factors of SoC and higher occupation on general health. We have also acknowledged the need for further research in this area, particularly regarding the association between general health status and other variables in the study.

Page 23-24 line 562-596:

*Interestingly, senior leaders in this study reported high general health status, contradicting wider study findings regarding low well-being and high work-related stress. How the term ‘general health’ was interpreted by senior leaders is unclear, this could be perceived as encompassing physical and/or psychological dimensions. It is plausible that whilst leaders in this study report work-related stress which impacts well-being, they may engage in health-promoting behaviours (e.g. physical activity), and thus perceive their health status as high. This data was not captured and requires further study, amongst a paucity of evidence regarding the presence of health-promoting behaviours in this population. Another mechanism may be through SoC, unpublished evidence from Sweden indicates the protective influence of SoC in reducing the impact of work-related stress on general health [83]. This is further supported by longitudinal research in the UK that suggests a salutogenic effect of strong SoC on chronic disease incidence [84]. Whilst associations between variables were not explored in this study, this warrants the need for further research to examine associations between perceived general health with other well-being and work-related indicators. Furthermore, although senior leaders in this study report lower well-being that the general population, perceived general health status is comparable to Census data collected in England and Wales in 2021 [85]. This is in line with research from France demonstrating comparable general health status between educational practitioners and other professions [86]. Senior leadership is considered a high occupational grade, this may offer protection for physical health as evidenced by findings in the Whitehall and Whitehall II cohort studies regarding occupation rank and psychosocial work environment [87,88].*

**General: Do you have more data on the retention rates of senior leaders in Wales and Northern Ireland? If so, that would reinforce the claims you make by a lot. Tying to actual burnout levels now would be great.**

We have included some data context to discussions around attrition, including available data from the Welsh Government regarding attrition. As specific breakdown by all senior leadership positions is not publicly available, we have included stats for secondary school leaders and educational practitioners more generally, to demonstrate attrition. This data is not publicly available in Northern Ireland. The following revisions include:

Page 20 line 475-483:

*Available data for Wales shows an increase in senior leaders from secondary schools leaving the profession over the last 10 years, this was highest in 2022 [24]. Whilst this data is only available for senior leaders in secondary schools, there has also been a steady increase in all teaching staff leaving the profession (e.g. n=414 in 2020, n=756 in 2022) [24]. Further, a survey of over 2,000 senior leaders across England, Wales and Northern Ireland suggests increasing dissatisfaction of school leadership as a career choice and fewer aspirations of those in deputy/assistant roles to progress to senior headship level [23]. Most recently, senior leaders in Wales and Northern Ireland have resorted to industrial action, citing workload, below-inflation pay awards and chronic school underfunding as key concerns within the profession [68,69][70]. This suggests these work-related pressures persist, and industrial action is ongoing at the time of writing [71].*

We have included additional references to new announcements of ongoing industrial action in Northern Ireland. We have also added a further citation, a report by the National Association of Head Teachers (2021), see citation 71. This report surveyed over 2,000 headteachers in England, Wales and Northern Ireland and findings further support concerns regarding attrition of senior leaders, and importantly, progression to top senior leadership roles from current assistant/deputy leaders.

**Page 16: Before breaking down each category, I recommend some broad, interesting findings in a short paragraph. Give the reader a taste, then expand upon those findings in each subsection (as you do). Remember, show the forest, then the trees! This is common practice.**

Thank you for this suggestion. Due to increased word count with added revisions, we feel this is sufficiently addressed within the first paragraph of the Discussion chapter which summarises the key findings:

Page 19 line 434-441:

*This study aimed to examine the working situation, well-being and work-related stress of senior school leaders in Wales and Northern Ireland and explore gender differences during a period of educational crisis leadership within the COVID-19 pandemic between 2021 and 2022. This is part of an ongoing international study through the COVID-HL network, with specific relevance to current and emerging policy and practice in Wales and Northern Ireland. Findings suggest that senior school leaders reported high workloads, low well-being, depressive symptoms and high work-related stress. Senior leaders also had high levels of exhaustion and psychosomatic complaints indicating burnout and were engaging in self-endangering working behaviours, these were significantly higher in female senior leaders.*

We feel that the majority of paragraphs begin with an overview of key findings for that outcome (e.g. see below). On occasions where the paragraph does not start with headline findings, we believe it important to maintain a flow in writing, by introducing the next discussion of findings with a linking sentence.

For example:

Page 20 line 461-462:

*The majority of senior leaders in this study reported low well-being, lower than the general adult population pre-pandemic [60] and COVID-HL international study data from Hong Kong [33,34] and Switzerland [35].*

Page 19 line 469-470:

*The large majority of senior leaders in this study reported a higher workload than before the pandemic and working at least 50 hours per week*

Page 21 line 495-496:

*In this study, senior leaders reported often giving up leisure activities in favour of work, sacrificing sufficient sleep and waiving breaks during the working day*
