# Supplementary material for: The well-being and work-related stress of senior school leaders in Wales and Northern Ireland during COVID-19 “educational leadership crisis”: A cross-sectional descriptive study
Source: PLoS One. 2024 Apr 10;19(4):e0291278. doi: 10.1371/journal.pone.0291278 (PMC11006137; doi:10.1371/journal.pone.0291278)
Supplement: Attachment — Submitted filename: Response to reviewers.docx [file pone.0291278.s008.docx]

**Response to reviewer**

We would like to express our sincere gratitude to Dr De Voto for a second round of suggested revisions, and again for the productive and positive comments regarding the manuscript. This review process has significantly improved the manuscript, and we are grateful for the time taken to provide such useful feedback.

Please find below a bullet point list of revisions followed by changes within the manuscript addressing these, including page and line number.

**Abstract**

- Sentence 2: this sentence read a little too long this early in the work. I would chop into 2 sentences/complete thoughts.

Page X line X

*Senior school leaders were at the forefront and were exposed to particularly high demands during a period of “crisis leadership”. This occupation were already reporting high work-related stress and large numbers leaving the profession preceding COVID-19.*

- I would spell out ‘HL’- Health literacy; I wanted to know right away who these folks were, even if brief.

Page X line X

*This cross-sectional descriptive study through the international COVID-Health Literacy*

**Introduction**

- The into is very well written, but I think the purpose of the study came a little late- about five pars. in. I recommend saying your study upfront, then going into all the great detail. This creates a nice ‘sign post’ for reader, easing any concerns.

We have included a sentence stating the main aim of this study in the second paragraph, following an initial background to the topic.

Page X line X

*Senior school leaders are responsible for all aspects of school life and therefore had to cope with high demands due to the COVID-19 pandemic, a period defined as “crisis leadership” [10,11]. This study aimed to examine the working situation, well-being and work-related stress of senior school leaders in Wales and Northern Ireland during this period of crisis leadership.*

**Methods**

- I would say right away your sample, then talk about your context. That way, we know right away. Just helps the reader not have to dig for it. In parentheses is fine in the first paragraph, then expound later (as you do).

Page X line X

*The COVID-19 School Principals Survey was administered to senior school leaders, recognised in this study as headteachers, deputy/assistant headteachers, or school staff with leadership and/or management responsibilities. The survey was conducted in Wales (n=172) between June and November 2021 and Northern Ireland (n=151) between March and May 2022.*

- Measure: I would say each measure first (in a few tight sentences), then talk about them as you do. Again, just helps the reader follow the progression.

Page X line X

Measures

*The 33-item COVID-19 School Principals Survey captured demographic characteristics in addition to the integration of adapted or standardized scales. These included work-related factors (weekly workload/teaching load), general health, well-being (WHO-5), work-related Sense of Coherence (SoC), exhaustion and psychosomatic complaints (short-form items from the Burnout Assessment Tool (BAT)), self-endangering behaviour (self-endangering work behaviour scale) and perceived stress (Perceived Stress Scale).*

- Was the SOC and WHO-5 part of the principal survey? This is a little unclear. Or are there multiple surveys used?

Thank you for drawing our attention to this, we have included a sentence addressing your previous comment regarding a brief paragraph about the measures that makes it clear that existing scales were integrated into the school principal survey.

Page X line X

Measures

*The 33-item COVID-19 School Principals Survey captured demographic characteristics in addition to the integration of adapted or standardized scales. These included work-related factors (weekly workload/teaching load), general health, well-being (WHO-5), work-related Sense of Coherence (SoC), exhaustion and psychosomatic complaints (short-form items from the Burnout Assessment Tool (BAT)), self-endangering behaviour (self-endangering work behaviour scale) and perceived stress (Perceived Stress Scale).*

**Results**

- I love how you match the measures in the methods described directly to the headers here; super helpful!

Thank you! We are glad this organisation is useful for the reader.

- Before diving right into the results, provide a little organizer paragraph. What are you planning on telling else? What are the key takeaways? Give us a quick snapshot. Again, just about guiding the reader along your journey.

Thank you for this useful suggestion, we have included an introductory paragraph at the start of the results section to summarise the key findings.

Page X line X

*Results*

*Findings from this study suggest that senior school leaders reported high workloads (54.22±11.30 hours/week), low well-being (65.2% n=202, mean WHO-5 40.85±21.57), depressive symptoms (WHO-5 34.8% n=108) and high work-related stress (PSS-10: 29.91±4.92). Senior leaders also had high levels of exhaustion (BAT: high/very high 89.0% n=285) and psychosomatic complaints (experiencing muscle pain 48.2% n=151) indicating burnout. Senior leaders in this study were engaging in self-endangering working behaviours (74.7% gave up leisure activities in favour of work, 63.4% sacrificed sufficient sleep), these were significantly higher in female senior leaders (work extensification males: 4.21 ± 0.62, females: 4.41 ± 0.50).*

**Discussion**

- Page 20: I would avoid the word ‘interesting’ when referring to data. Be a little more impartial on word choice.

Page X line X

*Furthermore, findings from the teaching profession in Switzerland reported that prolonging working hours mediates the relationship between work overload and exhaustion, thus, work extensification is used as a coping mechanism to high working demands [74].*

- Page 23, line 449: This sentence (another mechanism..) reads off.

Page X line X

*This high general health status reported by senior leaders in this study may also be explained through SoC, with unpublished evidence from Sweden indicating the protective influence of SoC in reducing the impact of work-related stress on general health [83].*

- Page 24, line 462: A lot of research in the US, AUS, and GER back this up. I would include/cite. Here are some examples from School Leadership & Management, an intl journal (Streipe et al., 2023; De Voto & Superfine, 2023; Beckmann & Domnique Klein, 2023)

Thank you for sharing this interesting international evidence, this has been added as below.

Page X line X

*International evidence has demonstrated the importance of organisational preparedness, ensuring capacities are in place across systems and settings to safeguard the well-being of those at the forefront of decision making and leadership [90]. This should be considered in times of crisis and periods of higher work-related demands requiring adaptations to roles and responsibilities [91].*
